# Editorial for the Special Issue on Advanced Materials, Structures and Processing Technologies Based on Pulsed Laser

**DOI:** 10.3390/mi12121507

**Published:** 2021-12-01

**Authors:** Youmin Rong, Congyi Wu, Yu Huang

**Affiliations:** 1 State Key Lab of Digital Manufacturing Equipment and Technology, Huazhong University of Science and Technology, Wuhan 430074, China; cyw@hust.edu.cn; 2 School of Mechanical Science and Engineering, Huazhong University of Science and Technology, Wuhan 430074, China

Pulsed lasers are lasers with a single laser pulse width of less than 0.25 s, operating only once in every certain time interval. Commonly used pulsed lasers are nanosecond, femtosecond, and picosecond lasers. A pulsed laser produces short pulses with a short interaction time with the material, which can largely avoid impact on the thermal movement of molecules and has a minimal thermal impact on the surrounding materials, thus, having significant advantages in precision microfabrication. It is now widely used in flexible electronics, chips, medicine, and other fields, such as photographic resin curing, microwelding, vision correction, heart stent manufacturing, etc. However, as an emerging processing technology, the application prospects of pulsed lasers have yet to be fully expanded, and there is still a need to continuously explore the mechanisms of interaction with materials, to manufacture advanced functional structures, and to develop advanced process technologies.

There are six papers published in this Special Issue focusing on pulsed laser machining. From metallic [1,2], inorganic [3] and polymeric [4,5] materials to biomass materials [6], from surface texture microstructures [1] and grain microstructures [2] to various types of functional contour structures [4,5], from the laser processing of metallic materials [1,2] and inorganic nonmetallic materials [3] to polymeric materials [4,5], from flexible electronics [4,5] and new energy batteries [1] to biomedicine [6], the articles in this Special Issue explore the specific applications of pulsed lasers in various fields.

In pulsed laser processing of metallic materials, Berhe et al. [1] studied the wettability behavior of structured and unstructured LiFePO4 electrodes. Firstly, the wettability morphology of the structured electrode was analyzed, and the electrode geometry as quantified in terms of ablation top and bottom width, ablation depth, and aspect ratio. From the result of the geometry analysis, the minimum measured values of aspect ratio and ablation depth were used as structured electrodes. Laser structuring with pitch distances of 112 μm, 224 μm, and 448 μm was applied. Secondly, the wettability of the electrodes was measured mainly by total wetting time and electrolyte spreading area. This study demonstrates that the laser-based structuring of the electrode increases the electrochemically active surface area of the electrode. The electrode structured with 112 μm pitch distance exhibited the fastest wetting at a time of 13.5 s. However, the unstructured electrode exhibited full wetting at a time of 84 s. Further, Fu et al. [2] studied the microstructure of a pulsed laser beam welded oxide dispersion-strengthened (ODS) eurofer steel. With a laser power of 2500 W and a duration of more than 3 ms, full penetration could be obtained in a 1 mm thick plate. Material loss was observed in the fusion zone due to metal vaporization, which can be fully compensated by the use of filler material. The solidified fusion zone consisted of an elongated dual phase microstructure with a bimodal grain size distribution. Nano-oxide particles were found to be dispersed in the steel. Electron backscattered diffraction (EBSD) analysis showed that the microstructure of the heat-treated joint was recovered with a substantially unaltered grain size and lower misorientations in different regions. The experimental results indicate that joints with fine grains and dispersed nano-oxide particles can be achieved via pulsed laser beam welding using a filler material and a postheat treatment. 

In the pulsed laser processing of inorganic nonmetallic materials, Wang et al. [3] presented a facile laser-based surface texturing technique to modulate and control the surface functionalities (i.e., wettability and hardness) of Zr-based BMG. Laser surface texturing was first utilized to create periodic surface structures, and heat treatment was subsequently employed to control the surface chemistry. The experimental results indicated that the laser textured BMG surface became superhydrophilic immediately upon laser texturing, and turned superhydrophobic after heat treatment. Through surface morphology and chemistry analyses, it was confirmed that the wettability transition could be ascribed to the combined effects of the laser-induced periodic surface structure and controllable surface chemistry. In the meantime, the microhardness of the BMG surface had been remarkably increased as a result of laser surface texturing. The facile laser-based technique developed in this work has shown its effectiveness in modification and control of the surface functionalities for BMG, and it is expected to endow more useful applications.

In the pulsed laser processing of polymeric materials, Xu et al.’s [4] studies on the thermoplastic PET film and on temperature field-assisted ultraviolet nanosecond (UV–ns) pulse laser processing of polyethylene terephthalate (PET) film were performed to investigate the photothermal ablation mechanism and the effects of temperature on laser processing. The results showed that the UV–ns laser processing of PET film was dominated by the photothermal process in which PET polymer chains decomposed, melted, recomposed and reacted with the ambient gases. The ambient temperature changed the heat transfer and temperature distribution in the laser processing. Low ambient temperature reduced the thermal effect and an increase in ambient temperature improved its efficiency. Following on further, Wu et al. [5] prepared a contact spacer for a flexible tactile sensor using a thermoset polymer PI film UV–nanosecond laser cutting process. Taking a three factor, five level orthogonal experiment, the optimum laser cutting process was obtained (pulse repetition frequency 190 kHz, cutting speed 40 mm/s, and RNC 3). With the optimal process parameters, the minimum diameter was 24.3 ± 2.3 μm, and the minimum HAZ was 1.8 ± 1.1 μm. By analyzing the interaction process between nanosecond UV laser and PI film, the heating–carbonization mechanism was determined, and the influence of the process parameters on the quality of a micro-hole was discussed in detail in combination with this mechanism. It provides a new approach for the quantitative industrial fabrication of contact spacers in tactile sensors.

In the pulsed laser processing of biomass materials, Michalik et al. [6] deals with the medical application of diode-lasers. A short review of medical therapies is presented, considering the wavelength applied, continuous wave (cw) or pulsed regimes, and their therapeutic effects. Special attention was paid to the laryngological application of a pulsed diode laser with wavelength 810 nm, and the dermatologic applications of a 975 nm laser working at cw and pulsed mode. The efficacy of the laser procedures and a comparison of the pulsed and cw regimes is presented and discussed.

I would like to take this opportunity to thank all the authors for submitting their papers to this Special Issue. I would also like to thank all the reviewers for dedicating their time and helping to improve the quality of the submitted papers.
